# Apatinib in refractory radiation-induced brain Edema: A case report: Erratum

**DOI:** 10.1097/MD.0000000000009589

**Published:** 2018-01-05

In the article, “Apatinib in refractory radiation-induced brain Edema: A case report”,^[1]^ which appeared in Volume 96, Issue 46 of Medicine, the authors’ degrees should have appeared as Wei Guo Hu MD, Yi Ming Weng MS, Yi Dong MS, Xiang Pan Li MD, Qi Bin Song MD.
